# How Do Induced Affective States Bias Emotional Contagion to Faces? A Three-Dimensional Model

**DOI:** 10.3389/fpsyg.2020.00097

**Published:** 2020-01-31

**Authors:** Andrés Pinilla, Ricardo M. Tamayo, Jorge Neira

**Affiliations:** ^1^Quality and Usability Lab, Institute of Software Engineering and Theoretical Computer Science, Faculty of Electrical Engineering and Computer Science, Technische Universität Berlin, Berlin, Germany; ^2^Laboratorio de Cognición Implícita, Departamento de Psicología, Universidad Nacional de Colombia, Bogotá, Colombia

**Keywords:** emotional contagion, facial expressions, evaluative space model, affective states, faces, emotions

## Abstract

Affective states can propagate in a group of people and influence their ability to judge others’ affective states. In the present paper, we present a simple mathematical model to describe this process in a three-dimensional affective space. We obtained data from 67 participants randomly assigned to two experimental groups. Participants watched either an upsetting or uplifting video previously calibrated for this goal. Immediately, participants reported their baseline subjective affect in three dimensions: (1) positivity, (2) negativity, and (3) arousal. In a second phase, participants rated the affect they subjectively judged from 10 target angry faces and ten target happy faces in the same three-dimensional scales. These judgments were used as an index of participant’s affective state after observing the faces. Participants’ affective responses were subsequently mapped onto a simple three-dimensional model of emotional contagion, in which the shortest distance between the baseline self-reported affect and the target judgment was calculated. The results display a double dissociation: negatively induced participants show more emotional contagion to angry than happy faces, while positively induced participants show more emotional contagion to happy than angry faces. In sum, emotional contagion exerted by the videos selectively affected judgments of the affective state of others’ faces. We discuss the directionality of emotional contagion to faces, considering whether negative emotions are more easily propagated than positive ones. Additionally, we comment on the lack of significant correlations between our model and standardized tests of empathy and emotional contagion.

## Introduction

Emotions frequently involve reactions to events and people, influencing in turn our judgments about the affective states of others. Various prominent theoretical frameworks (Hatfield et al., 1993, 1994; Forgas, 1995; Peters and Kashima, 2015) comprise at least one type of psychological mechanism by which humans and other mammalians automatically converge to the affective states of their peers when it is socially appropriate (de Waal, 2012; Palagi et al., 2015). This mechanism has been defined as “a tendency to automatically mimic and synchronize expressions, vocalizations, postures and movements with those of another person’s, and, consequently, to converge emotionally” (Hatfield et al., 1994, p. 5).

Evidence of automatic affective convergence for facial mimicry (Dimberg, 1982; Dimberg et al., 2000; Dimberg and Thunberg, 2012; Varcin et al., 2019), vocalizations (Cappella and Planalp, 1981; Fujiwara and Daibo, 2016) and body postures (Condon and Ogston, 1966; Schmidt et al., 2011), support the existence of an automatic mechanism for emotional contagion. Of course, automatic transmission of affect among dyads (Bruder et al., 2012; Butler, 2015) and beyond (Dezecache et al., 2013; Kramer et al., 2014) is not the only psychological process that modulates people’s affective responses. Clearly, additional mechanisms linked to individual differences (Doherty, 1997; Künecke et al., 2014), specific appraisals (Fontaine et al., 2007), pragmatic goals (Fischer and Hess, 2017) and social roles (Murray, 1933) mediate the propagation of emotions. In fact, previous work has significantly contributed to clarify basic concepts in the field. For instance, clear differences exist today between deeply related concepts such as mood, affect, and emotion (Forgas, 1995; Russell, 2003; Peters and Kashima, 2015) and between related explanatory frameworks such as affect diffusion, emotional contagion, and affect infusion (Dezecache et al., 2015; Peters and Kashima, 2015).

However, to our knowledge, no specific framework exists to quantify the amount of subjective emotional contagion among individuals. Usually, emotional contagion is assumed by observing whether participants’ affective responses (i.e., subjective ratings) or implicit automatic reactions (i.e., facial electromyographic responses) converge to the valence of previously presented stimuli (e.g., faces, videos, sounds, scenes, actors, or peers). In our view, an ideal framework for quantifying emotional contagion should describe how much affect is transferred from the participant’s immediate emotional context to subsequent emotional responses.

We believe that such a model could contribute to understand the interaction between explicit and implicit cognitive mechanisms involved in emotional contagion. The need for a specific mathematical model for quantifying subjective emotional contagion might be illustrated by contemporary research concerned with the influence of automatic facial mimicry responses – predominantly related to implicit mechanisms – on subjective and affective ratings – predominantly related to explicit mechanisms – (e.g., Fujimura et al., 2010; Dezecache et al., 2013; Sato et al., 2013; Künecke et al., 2014; Deng and Hu, 2018). For instance, Deng and Hu (2018), collected subjective ratings of valence in a single bipolar scale together with electromyography (EMG) recordings from zygomaticus mayor (ZM) and corrugator supercilii (CS) muscles, from participants exposed to positive (happy) and negative (angry) faces. In two experiments, the authors found a lack of correlation between ZM activity and subjective ratings in response to positive stimuli. Similarly, in a conceptually analogous manipulation of transitive affect, Dezecache et al. (2013) found that explicit identifications of positive faces were not above chance, while ZM activity increased for positive faces (Dezecache et al., 2013). Furthermore, results from two experiments conducted by Fridlund (1991) support the claim that implicit facial expressions may be erratically associated with explicit subjective reports of emotional experience – for a detailed review, see Barrett et al. (2019). We believe these results are puzzling because subjective measures either collected in a single bipolar scale (Deng and Hu, 2018) or as discrete identifications (Dezecache et al., 2013) do not provide enough information to determine the amount of subjective contagion experienced by participants. In our view, research could benefit from disentangling the amount of contagion triggered by the stimuli from the amount of contagion that bias further affective judgments, pondering that a single bipolar measure of valence might not fully describe the subjective and affective processes of emotional contagion experienced by individuals. For instance, as suggested in the evaluative space model (ESM) (Cacioppo et al., 1997), increases in negative affect are not necessarily proportional to decreases in positive affect, and baseline subjective responses to positive stimuli usually offset baseline activation for negative stimuli. Therefore, using a single bipolar measure of valence as an index of subjective affect could have obscured genuine correlations between automatic mimicry and subjective affect in previous studies.

In the present paper, we propose a simple mathematical model to quantify emotional contagion to faces by computing the distance between two emotional coordinates in a three-dimensional affective space. The first coordinate represents the affective state that participants subjectively self-reported after observing a video. The second coordinate represents subjective judgments about the affective state of unfamiliar faces. Given that people tend to ascribe aspects of their own affective state to others (Forgas, 1995; Van Boven and Loewenstein, 2003), we assume that the distance between the first and second coordinates, indexes the amount of emotional contagion to faces experienced by participants. This is, how much convergence is there between participant’s affective states before (T1) and after seeing the faces (T2). The measure at T1 is the evaluation of the video and the measure at T2 is the evaluation of each face. We interpret participant’s evaluation of faces as an index of their affective state after watching each face, based on previous evidence suggesting that evaluation of other’s facial expressions correlates with self-reported affective states (Blairy et al., 1999). Additional evidence shows that activation of the amygdala during a face evaluation-task correlates with participant’s subjective evaluations of angry faces (Nomura et al., 2004). Therefore, subjective judgments about the affective state of other’s faces can be considered an index of participants’ affective state. This evidence is in turn consistent with predictions from the Affect as Information mechanism proposed in the Affect Infusion Model (AIM) (Forgas, 1995), which suggests that people tend to use their own emotional state as heuristic to attribute emotions to others.

In short, we propose a model to quantify the distance between two different *subjective evaluations*. The first one, induced by a video, the second one, involved in the evaluation of unfamiliar faces. In other words, the model calculates the distance between participants’ self-reported affective state (triggered by the video) and the influence exerted by this affective state on the emotional contagion to unfamiliar faces. Shorter distances indicate more emotional contagion to faces and larger distances indicate the opposite.

Computing the shortest distance between these two affective states presupposes an efficient psychological mechanism for emotional contagion based on *minimal effort* (Forgas, 1995) or *energetic efficiency* (Cacioppo et al., 1997). This mechanism is conceived as an organism’s tendency to adopt the shortest path and least effortful strategy to yield a subjective affective response, if it satisfies minimal adaptive requirements. We preferred a three-dimensional affective space based on monopolar ratings similar to the one suggested by Cacioppo et al. (1997) for two reasons. Firstly, because contemporary evidence suggests that a three-dimensional model does not exclude a bi-dimensional one (Mattek et al., 2017) and that monopolar measures do not preclude additional affective dimensions (e.g., dominance in the first case or net predisposition in the second case). Second, a three-dimensional space maps well into commonly used psychophysiological measures such as EMG activity from ZM and CS muscles for positivity and negativity, respectively, and electrodermal activity (EDA) or heart rate variability (HRV) for arousal (Bernat et al., 2006).

To extend the measurement framework for subjective responses of emotional contagion, we designed an experiment in which participants were induced to either a positive or a negative affective state by watching an affectively laden video clip. Immediately, we assessed their baseline subjective emotional state by asking them to self-report how the video made them feel in three dimensions: positivity, negativity, and arousal. In a second phase, participants were asked to judge the affective states of unfamiliar faces using the same three dimensions. As mentioned above, given that people tend to use their own affective states as a heuristic to judge the affective state of others (Forgas, 1995; Van Boven and Loewenstein, 2003), we reasoned that participant’s evaluations of faces could index the amount of emotional convergence between the affective state exerted by the video and the affective state associated to each face. If emotional contagion is automatic and transitive (Dezecache et al., 2013), then it should facilitate emotional convergence by spreading the affective states triggered by the video to the affective state involved in the participant’s judgments of unfamiliar faces. Therefore, based on the principle of least effort, we hypothesized that participants induced to a negative affective state would show more emotional contagion to angry than happy faces, while participants induced to a positive affective state would show more contagion to happy than angry faces.

Finally, we wanted to explore the relationship between our suggested measure of subjective emotional contagion and key psychometric measures of emotional contagion and empathy. Consequently, participants responded to the Emotional Contagion Scale (ECS; Doherty, 1997; Gouveia et al., 2007), the Interpersonal Reactivity Index (IRI; Davis, 1980; Pérez-Albéniz et al., 2003), the Questionnaire of Prosocial Conduct (QPC; Martorell et al., 2011), and the Basic Empathy Scale (BES; Jolliffe and Farrington, 2006; Merino-Soto and Grimaldo-Muchotrigo, 2015).

## Materials and Methods

### Participants

Sixty-five undergraduate students from the National University of Colombia participated in the study. Their average age was 21.6 years old (*SD* = 3.7); 50.7% were women. Thirty-two participants were randomly assigned to the negative induction group and 35 to the positive induction group. Boxplots revealed outliers for two participants, whose data were excluded from further analysis. All participants provided written informed consent prior to participating in the experiment, in accordance with the ethical principles of the Declaration of Helsinki.

### Materials

Inquisit 4 software was used to record subjective ratings and present all instructions, surveys and prompts on a 23-inch (1920 × 1980) computer screen. The approximate physical distance between the participant and the screen was 20 inches. Two scenes from the FilmStim (Schaefer et al., 2010) were used for emotional induction: *Dead Poets Society*^1^ and *American History X*. Twenty images of the Pictures Of Facial Affect (POFA; Ekman, 1993) were used (10 happy and 10 angry). Affective responses to these stimuli and a similar set of stimuli were calibrated for the present population (see repository with Supplementary Material^2^). Additionally, the ECS (Doherty, 1997; Gouveia et al., 2007) was used to estimate the correlation between the proposed measurement model with individual differences. Finally, three empathy questionnaires adapted to Spanish were used: IRI (Davis, 1980; Pérez-Albéniz et al., 2003), QPC (Martorell et al., 2011), and BES (Jolliffe and Farrington, 2006; Merino-Soto and Grimaldo-Muchotrigo, 2015)^3^.

### Procedure

The experiment had four phases. In the first phase, participants were induced to either negative or positive affective states by watching a positive or a negative video clip taken from the FilmStim database (Schaefer et al., 2010) according to the randomly assigned experimental group. Immediately after watching the video, the following question was presented to participants: “How did this scene make you feel?” There were three Likert scales bellow the question. The scales had one label at each side and ranged from 0 to 100. In the scale that measured the negativity dimension, the labels were “0 – not bad at all,” and “100 – very bad”; in the scale that measured the positivity dimension, the labels were “0 – not good at all,” and “100 – very good”; in the scale that measured the arousal dimension, the labels were “0 – not restless at all,” and “100 – very restless.” In the present article, all prompts and instructions were translated from Spanish. To maximize participants’ attention to the questions, their order randomly changed for each trial. The labels were red for the negativity dimension, green for the positivity dimension, and blue for the arousal dimension. The colors of labels remained constant throughout the experiment and were used to help participants discriminate the dimension to evaluate in each prompt.

In the second phase, 20 images of faces taken from the POFA were shown in random order for each participant (10 positive and 10 negative). The randomization compensated possible carryover effects produced by the faces. Before each image, a gray screen with a white cross in the middle was shown for 1 s. Every face was shown for 3 s. After each face, three questions with the same statement were shown. The statement was: “How do you think this person feels?” Participants answered using three Likert monopolar scales, just like the ones presented after the video. The scales had two labels (one in each side). The labels were different for each question. In the question that measured the negativity dimension, the labels were “0 – not bad at all,” and “100 – very bad”; in the question that measured the positivity dimension, the labels were “0 – not good at all,” and “100 – very good”; and in the question that measured the arousal dimension, the labels were “0 – not restless at all,” and “100 – very restless.”

In the third phase, participants were asked if they had previously seen the video clip from FilmStim (Schaefer et al., 2010), and two surveys used for the creation of the FilmStim (Schaefer et al., 2010) were applied as a manipulation check. These were the Positive and Negative Affect Schedule (PANAS) (Watson et al., 1988) and a self-reported emotional arousal scale (Schaefer et al., 2010).

Finally, in the fourth phase, participants were asked to answer the questionnaires in the following order: IRI, QPC, BES, and ECS.

### Emotional Contagion Equation

We hypothesized that exposition to positive and negative videos could bias emotional contagion to happy versus angry faces. Therefore, we estimated the shortest distance between two affective coordinates (video and face) in a three-dimensional Euclidean space. The emotional distance between the affective state induced by the video and the subjective evaluation of the faces can be expressed using the equation:

(1) $$\mathrm{ec}=1-\left(\frac{\sqrt{{\left(n_{v}-n_{f}\right)}^{2}+{\left(p_{v}-p_{f}\right)}^{2}+{\left(a_{v}-a_{f}\right)}^{2}}}{100\times \sqrt{3}}\right)$$

where *n*_*v*_, *p*_*v*_, and *a*_*v*_ represent participants’ affective state induced by the video in the negativity, positivity, and arousal dimensions, respectively, and *n*_*f*_, *p*_*f*_, and *a*_*f*_ represent participants’ evaluation of the faces in the same dimensions. The value inside the parenthesis is subtracted from one because (a) it represents emotional distance analogously to the ESM (Cacioppo et al., 1997) and (b) because we assume that emotional contagion to faces is the inverse of this distance. To facilitate data analysis, we rescaled values to a range from 0 to 1. Thus, we divided everything inside the square root by the maximum possible distance between the two affective coordinates.

## Results

Data were analyzed using a 2 × 2 mixed ANOVA, with negative and positive emotional induction as between-group factor, happy and angry faces as within-group factor. The dependent variable was the magnitude of emotional contagion to faces, as defined in Eq. 1. Outliers were detected in two participants whose data were excluded from further analysis. Data were normally distributed for all cells of the experimental design, as assessed by Shapiro–Wilk test (*p* > 0.05). The assumption of homogeneity of variances was met, according to Brown–Forsythe test (*p* > 0.05). A statistically significant two-way interaction was found, *F*(1, 65) = 103.957, *p* < 0.001, $\eta_{\text{p}}^{2}$ = 0.615. A main effect for type of face was found, *F*(1, 132) = 4.766, *p* = 0.031, $\eta_{\text{p}}^{2}$ = 0.035. A paired-samples *t*-test was conducted to compare the magnitude of emotional contagion to happy and angry faces in each group. A Bonferroni correction was applied. Emotional contagion was significantly greater for angry (*M* = 0.76, *SD* = 0.08) than happy faces (*M* = 0.41, *SD* = 0.18) in the negative group, *t*(31) = , *p* < 0.001. The opposite tendency was found in the positive group, where emotional contagion was significantly lower for angry (*M* = 0.54, *SD* = 0.15) than for happy faces (*M* = 0.71, *SD* = 0.13), *t*(34) = , *p* < 0.001. No main effect for type of emotional induction was found, *F*(1, 65) = 3.353, *p* = 0.072, $\eta_{\text{p}}^{2}$ = 0.049.

We were interested in analyzing the effect of gender on emotional contagion to faces. This required a hierarchical model because our sample size differed between each group (Huta, 2014, p. 21). Therefore, a linear mixed effects model was run to analyze whether gender had an effect on emotional contagion to faces. The fixed effects of the model where type of emotional induction and gender. The random effects were the intercepts for participants and type of face, as well as by-participant and by-type-of-face random slopes for the effect of emotional contagion. This model was compared to another model that had the same parameters but did not have a fixed effect for gender. Results of both models were compared using a Chi-squared test. Given that no significant differences were found, we infer that participant’s gender had no significant effect on emotional contagion to faces (χ^2^(1) = 1.68, *p* = 0.195).

Responses about familiarity with the videos were recorded for only 42 participants, due to a technical problem. Therefore, we excluded this question from the analysis. Otherwise, the manipulation check was successful. Overall, participants’ evaluations (see Table 1) were similar to the scores reported in the FilmStim (Schaefer et al., 2010).

**TABLE 1:** Summary of responses in the manipulation check and reported scores in the FilmStim (Schaefer et al., 2010) for the two videos used in the experiment.

	**Experiment score**	**FilmStim score**
*American History X*		
PA (positive affect)	2.55	2.04
NA (negative affect)	2.45	2.73
Arousal	5.44	5.84
*Dead Poets Society*		
PA (positive affect)	3.01	2.82
NA (negative affect)	1.56	1.21
Arousal	4.8	5.66

*Positive and negative affect are measured using a five-point scale, taken from the PANAS (Watson et al., 1988), while arousal is measured using a seven-point scale, proposed by the authors of the FilmStim (Schaefer et al., 2010).*

Results from the questionnaires were compared with the emotional contagion ratings. We predicted that participants with higher emotional contagion ratings would be more empathetic, as assessed with the empathy questionnaires, or more prone to emotional contagion, as assessed with the ECS (Doherty, 1997). However, we did not find this result (see Table 2). A Pearson correlation was performed between emotional contagion to faces and each questionnaire separately. No Bonferroni correction or similar was applied. There was a significant negative correlation between emotional contagion toward happy faces in the negative induction group and the IRI scores, *r*(30) = –0.40, *p* = 0.02 and ECS scores (Doherty, 1997), *r*(30) = –0.43, *p* = 0.01. In all the other cases correlations were not significant.

**TABLE 2:** Summary of Pearson correlations between questionnaires (*p*-values in parenthesis) and emotional contagion assessed with Eq. 1.

**Questionnaire**	**Negative induction group**		**Positive induction group**	
		
	**Happy faces**	**Angry faces**	**Happy faces**	**Angry faces**
ECS	−0.43 (0.015)*	−0.28 (0.127)	−0.28 (0.101)	−0.02 (0.929)
IRI	−0.40 (0.024)*	−0.18 (0.330)	−0.21 (0.219)	−0.04 (0.809)
BES	−0.30 (0.096)	−0.30 (0.099)	−0.10 (0.549)	−0.10 (0.559)
QPC	−0.19 (0.307)	−0.26 (0.148)	−0.23 (0.180)	−0.08 (0.658)

*p < 0.05.

## Discussion

Our findings suggest that participants minimized the emotional distance between the affective state triggered by the video and the affective state triggered by unfamiliar faces. When induced to a negative affective state, participants judged both angry and happy faces closer to a negative affective state. Conversely, when induced to a positive affective state, participants judged both happy and angry faces closer to a positive affective state. Plainly, participants’ induced affective state biased their emotional contagion to unfamiliar faces. Additionally, the results also show a double dissociation, happy and angry faces were differently evaluated by participants previously exposed to either an uplifting or an upsetting video.

**FIGURE 1:**
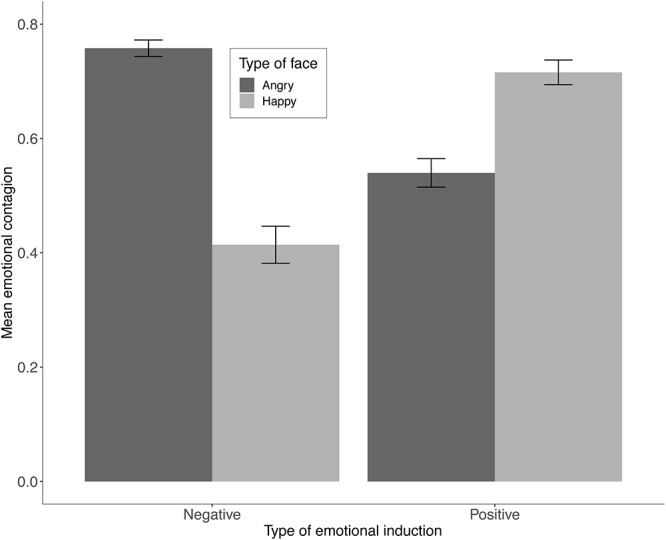
Mean emotional contagion toward angry and happy faces for the groups exposed to a negative and a positive video clips, as assessed with Eq. 1. Error bars depict 95% CI.

In our view, these results show key differences in the way emotional contagion operates, when it is triggered by negative versus positive emotions. Negative affective states generate more bias than positive states. This can be observed in Figure 1, where the highest emotional contagion is observed for the negative induction group toward angry faces. This finding is consistent with previous research suggesting that negative affective states trigger more behavioral changes than positive ones (Cacioppo et al., 1997).

In this experiment, we used two types of measures to assess participants’ affective states. A direct measure for the video and an indirect measure for the faces. The first was *direct* in the sense that participants were asked explicitly about their feelings after watching the video (“How did this scene make you feel?”). The second was *indirect* in the sense that participants were asked to rate the feelings they perceived from the faces (“How do you think this person feels?”). We used this later measure to estimate the amount of convergence between the affective states before and after the presentation of faces. As mentioned above, we decided to use an indirect (implicit) measure because (1) people tend to use their own affective state as a heuristic to judge others’ affective states (Forgas, 1995; Van Boven and Loewenstein, 2003) and (2) an indirect measure potentially reduces the noise caused by individual explicit preferences. For instance, if the second question would have been a direct measure, i.e., “how does this person make you feel?” the corresponding answers might have been biased by personal liking, and metacognitive judgments about the self. Similarly, if the question were “how do you feel?” the corresponding answer would imply a permanent higher-order judgment about the self and not a temporary automatic influence exerted by video on the judgment of each face. This methodological approach was inspired by previous research suggesting that people tend to misattribute the sources of their affective states, and that implicit measures tap earlier emotional processing-stages than direct measures do (e.g., Payne et al., 2010; Payne and Lundberg, 2014). Therefore, if emotional contagion involves basic and early emotional processing-stages as originally suggested by Hatfield et al. (1993, 1994), then there are more chances to measure it by indirectly estimating the influence it exerts on implicit (indirect) measures.

Our results provide independent support to the claim that emotional contagion is not always a linear process (Dezecache et al., 2015). Negative emotions seem to propagate more than positive emotions, and other people’s affective states are perceived more negatively when the observer is also in a negative affective state. This suggests that stronger emotional contagion occurs when a person, in a negative affective state, observes someone in a similar affective state.

This is interesting because previous research (Kramer et al., 2014) suggests that Facebook users tend to display more positive emotions when exposed to positive emotional content and more negative emotions when exposed to negative emotional content. Our results are consistent with these findings as well. Therefore, increasing exposition to negative emotional content might intensify negative emotions in the observer, which in turn increases sensitivity to emotional contagion to negative stimuli. Conversely, increasing exposition to positive emotional content might intensify positive emotions in the observer, magnifying emotional contagion to positive stimuli.

On the other hand, we did not find systematic correlations between psychometric questionnaires and our emotional contagion measure. In most cases, significant correlations were absent. In two cases where significant correlations were present, they were negative, suggesting that people displaying higher levels of empathy rated lower in our emotional contagion measure. However, the fact that these correlations were significant only for happy faces in the negative group (see Table 2) implies that those results cannot be generalized to all emotional contagion processes. In our view, these results are not completely surprising because questionnaires tap into a very different psychological process related to emotional contagion. Questionnaires are conceived as a personality-trait measure influenced by stable metacognitive judgments about the self in social situations. They are mainly based on explicit propositional knowledge (i.e., “I sometimes feel helpless when I am in the middle of a very emotional situation”; Davis, 1980). Instead, our measuring framework directly taps into specific momentarily states of emotional contagion hardly assessed by rationalized judgments about the self.

Based on our study, future experiments might investigate whether participants’ affective states influence automatic imitation of facial expressions. Do people imitate negative faces faster or with greater accuracy when induced to a negative affective state? We believe that the answer would be affirmative. In those experiments, automatic emotional responses could be assessed using electrophysiological signals. Previous research points out that activation of CS and ZM muscles are associated with negative and positive emotions, respectively (Dimberg, 1982). Additional research has found that activation of the sympathetic system is associated with HRV (Appelhans and Luecken, 2006). Thus, activity of the CS-muscle, ZM-muscle, and HRV would be equivalent to the negativity, positivity, and arousal dimensions used in our study, which are similar to the dimensions of the ESM (Cacioppo et al., 1997). However, it is important to consider that facial expressions might not always correlate with self-reported subjective affective states (Fridlund, 1991). Yet, the magnitude of the dissociations between subjective (i.e., participant’s ratings of positivity, negativity and arousal) and objective measures (e.g., EMG and HRV) could be assessed using a mathematical approach similar to Eq. 1. This approach would consist on calculating the emotional distance between subjective and objective emotional responses. This model would analyze individual differences in the magnitude of these dissociations, which in turn could help to quantify the relative contribution from each type of measure to the final affective state.

In short, our study provides evidence suggesting that people induced to a positive affective state show higher levels of emotional contagion to positive faces, while people induced to a negative affective state show higher levels of emotional contagion to negative faces. Furthermore, we provide evidence suggesting that subjective biases induced by current affective states are easily estimated by a simple mathematical model mapped onto a three-dimensional affective space. However, in scenarios where these two affective coordinates are similar due to factors not directly related to emotional stimulation (e.g., both states are neutral), the output of the model would indicate high emotional contagion, regardless of how much emotional change has been actually produced. This is a boundary condition of our model, which is exclusively useful for conditions where emotional contagion processes are reasonably assumed to be in operation.

Finally, the main contributions of this study are (1) to provide a measurement framework to analyze how affective states influence the directionality of emotional contagion and (2) to propose a methodological approach to analyze emotional contagion, not only as a binary outcome, but as a continuous quantitative variable.

## Author’s Note

The dataset used in this article was previously used in the Master’s Thesis of AP (Pinilla, 2017). This work was supervised by RT. The thesis is available at: http://bdigital.unal.edu.co/59467/1/1020759173.2017.pdf.

## Data Availability Statement

The datasets generated for this study are available at https://osf.io/52uj8/.

## Ethics Statement

Ethical review and approval was not required for the study on human participants in accordance with the local legislation and institutional requirements. The participants provided their written informed consent to participate in this study.

## Author Contributions

RT and AP proposed the experimental design. JN worked on acquisition of the data. All authors analyzed and interpreted the results.

## Conflict of Interest

The authors declare that the research was conducted in the absence of any commercial or financial relationships that could be construed as a potential conflict of interest.

## Footnotes

1 The FilmStim database offers two video clips taken from *Dead Poets Society*. We used the second one.

2 Videos and faces were quantitatively analyzed according to the assumptions of ESM (Cacioppo et al., 1997) during previous experiments. Datasets are available at: https://osf.io/52uj8/.

3 Questionnaire responses were analyzed and made available at: https://osf.io/52uj8/.
