# Supplementary material for: How Do Induced Affective States Bias Emotional Contagion to Faces? A Three-Dimensional Model
Source: Front Psychol. 2020 Jan 31;11:97. doi: 10.3389/fpsyg.2020.00097 (PMC7006022; doi:10.3389/fpsyg.2020.00097)
Supplement: Supplementary file 1 [file Table_1.docx]

Supplementary Material

Supplementary Table 1

Summary of correlations between questionnaires (p values in parenthesis).

|  | ECS | IRI | BES | QPC |
| --- | --- | --- | --- | --- |
| ECS |  | .7 (.000)** | .74 (.000)** | .45 (.000)** |
| IRI | .7 (.000)** |  | .6 (.000)** | .5 (.000)** |
| BES | .74 (.000)** | .6 (.000)** |  | .33 (.000)** |
| QPC | .45 (.000)** | .5 (.000)** | .33 (.000)** |  |

** *p < .001*
